# Resource management strategies for prioritizing non-scheduled surgical procedures in a tertiary public hospital

**DOI:** 10.1016/j.clinsp.2024.100482

**Published:** 2024-08-29

**Authors:** Marcelo Cristiano Rocha, Rafaela Alkmin da Costa, Edivaldo Massazo Utiyama

**Affiliations:** a Faculdade de Medicina da Universidade de São Paulo, São Paulo, SP, Brazil; b Hospital das Clínicas da Faculdade de Medicina da Universidade de São Paulo, São Paulo, SP, Brazil

Healthcare systems worldwide face the perennial challenge of balancing patient demand with finite resources, a struggle exacerbated in tertiary hospitals like the Hospital das Clínicas da Faculdade de Medicina da Universidade de São Paulo (HC-FMUSP). As a leading referral center serving millions in São Paulo and beyond, HC-FMUSP confronts the complex task of managing non-scheduled surgical procedures amidst high clinical acuity and resource variability.^1^

At Instituto Central of HC-FMUSP, the demand for emergency surgical services is intense, with over 42,000 emergency visits annually and approximately 280 non-scheduled surgeries per month. This volume underscores the critical need for efficient resource allocation, particularly in the operation theaters available around the clock. Historically, decisions regarding surgery prioritization were decentralized, relying heavily on individual physician judgment. This approach led to inconsistencies, inefficiencies, team discord and challenges in surgical planning, impacting patient care and staff satisfaction.

The National Confidential Enquiry into Patient Outcome and Death (NCEPOD) introduced a classification system categorizing surgeries by urgency ‒ IMMEDIATE, URGENT, EXPEDITED, and ELECTIVE ‒ based on clinical severity and need for immediate intervention. This framework provides clarity and consistency in prioritizing surgeries critical to patient survival and organ function preservation.^2^^,^^3^

Similarly, the World Society of Emergency Surgery (WSES) proposed the Timing of Acute Care Surgery (TACS) classification, which categorizes surgeries based on time frames ‒ ranging from immediate to over 48 hours ‒ according to clinical urgency. These frameworks not only standardize decision-making but also streamline communication among multidisciplinary teams, enhancing operational efficiency and patient outcomes.^1^^,^^4^^,^^5^

Regarding patient security and management tools in health services, the safety huddle is a daily multi-professional meeting in healthcare, which directs resource allocation by addressing daily demands and risks. A review of 158 studies showed positive impacts of performing safety huddles on team processes (67.7 %), including efficiency and communication (64.4 %), situational awareness (44.6 %), and team satisfaction (29.7 %). Approximately 44.3 % of studies reported improved clinical outcomes, like timeliness and reduced errors (31.4 %), and adverse events (24.3 %), benefiting overall patient care (20.0 %).^6^

In this way, Kanban, is a visual management tool derived from the Japanese word “card”, that enhances productivity by organizing workflow stages. Implemented in a Brazilian hospital, it notably reduced patient stay durations and eliminated bed availability issues.^7^

In response to the challenges at HC-FMUSP, a systematic approach to surgical prioritization was developed. Initially, a consensus was sought among 17 specialist physicians in General and Trauma Surgery regarding the priority of 50 real surgical cases. Results showed significant agreement, with priorities ranging from level 1 (highest urgency) to level 4 (lowest urgency) across cases. The intraclass correlation coefficient of 0.959 (95 % CI 0.937‒0.975, p < 0.001) indicated strong consensus among evaluators.

Parameters for surgical prioritization were then systematically outlined, focusing on intrinsic factors such as surgery criticality, impact on disease progression, and patient wait time, as well as extrinsic factors like concurrent surgical workload, procedure duration, and postoperative bed availability. Specialties collaborated to define time intervals for case resolution: immediate (within 1-hour), urgent (1‒6 hours), expedited (6‒24 h), semi-urgent (24‒48 h), and elective (over 48 h). This structured approach ensured that each specialty categorized procedures, accordingly, optimizing resource allocation and enhancing patient care coordination.

Following the establishment of a prioritization algorithm, a form was devised for surgical teams to complete at the time of operating room requests. This form categorizes procedures into six priority levels, from urgent (within 1-hour) to elective (more than 48 h), adaptable on the basis of changing patient needs and waiting time exceeding the expected. Each priority level is color-coded for visual clarity: red (priority 1) to gray (priority 6). A shared electronic panel disseminates this prioritized list among all surgical specialties, enhancing transparency and efficiency in resource allocation. This panel also includes the status of the operating surgical rooms, the surgical specialty responsible for the indication, the patient's age, the time they have been waiting for surgery, their bed, and their status in the queue (“waiting for the call”, “calling now”, “in procedure”, “procedure completion”, “awaiting destination bed”, “Post anesthesia recuperation”) as well as options for signaling pause, cancellation, or completion of the procedure, for updating patients in the queue (Fig. 1).

**Fig. 1:**
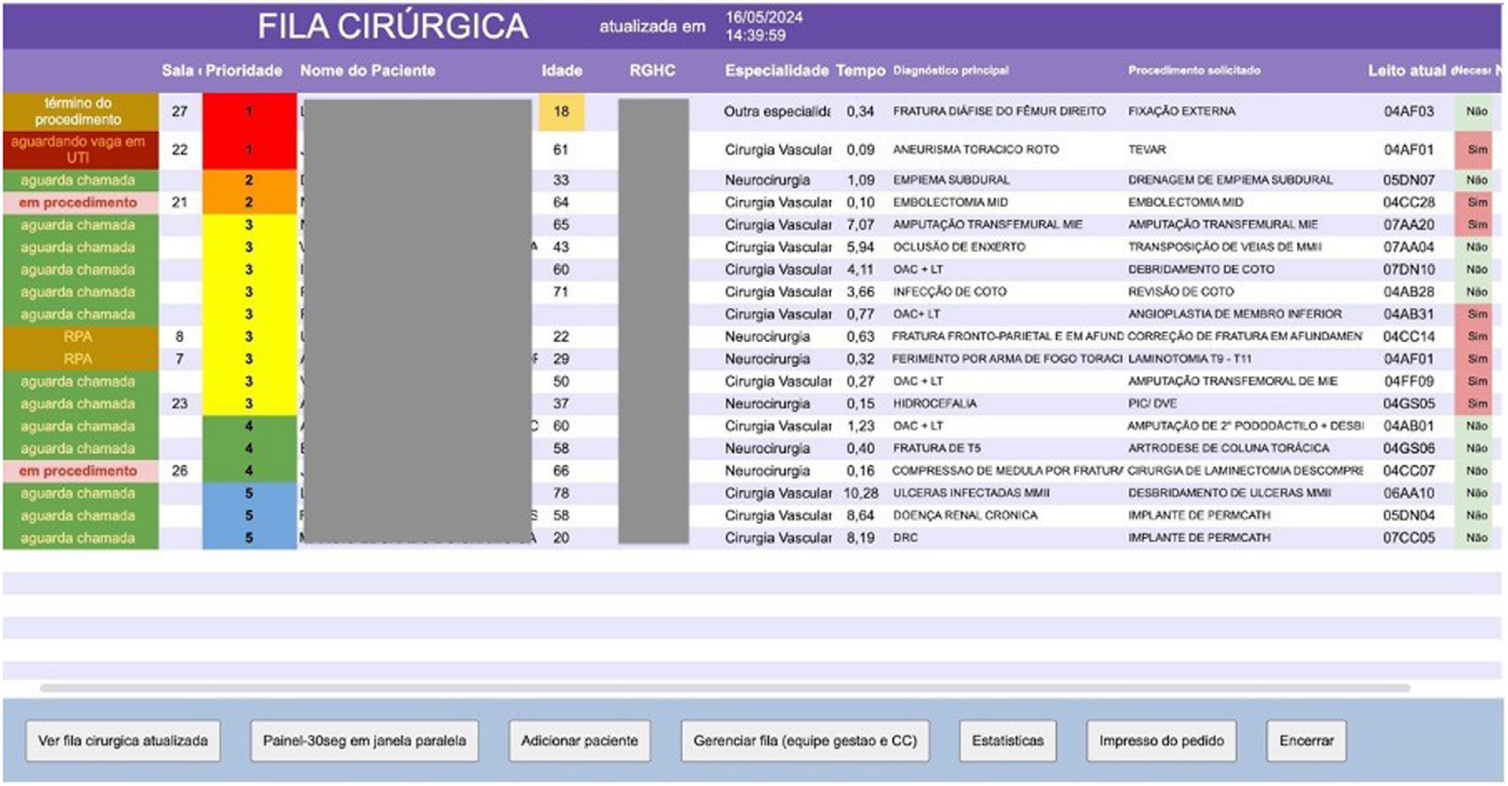
Shared panel with the list of patients waiting for an operating room.

A daily meeting was established with teams involved in surgical resolution, including surgeons from various specialties, the Surgical Center team (anesthesia and anesthesia supervision, nursing, and nursing management), the Referred Emergency Unit team (medical coordination and nursing), Nutrition, Supplies, and Medical Staff Management. At this meeting, the sequence of procedures to be performed that day is defined, considering intrinsic and extrinsic factors, evolving clinical needs, and resource constraints.

Initially tested without algorithmic guidance, subsequent implementation showed 81 % adherence to suggested priorities among 134 procedures deliberated in the first month. Therefore, the panel was implemented and dynamically updated with priorities and statuses, aiding daily surgical planning discussions involving surgeons, anesthesia teams, nursing, and emergency unit coordinators. This structured approach at HC-FMUSP optimizes surgical scheduling, minimizes delays, and enhances patient care coordination through systematic priority management and real-time updates.

Since its implementation, the prioritization system has yielded promising results. Preliminary data from 2023 indicate that over 80 % of the 2,508 non-scheduled surgeries were performed within the recommended timeframes, demonstrating improved workflow efficiency and resource utilization. Patients categorized as higher priority experienced significantly shorter wait times, aligning with clinical urgency guidelines. The waiting time for the procedure for each category during the evaluated period is described in Table 1.

**Table 1:** Waiting time for unscheduled surgical procedure, according to the ideal time limit for surgical intervention. HC-FMUSP, 2023 (n = 2508).

**Time limit for procedure**	**n**	**Median (hours)**	**Interquartile Range (hours)**
Up to 1 hour	340	1.26	[0.54‒2.43]
Between 1 and 6 hours	597	4.90	[2.12‒9.38]
Between 6 and 24 hours	736	16.62	[7.42‒30.53]
Between 24 and 48 hours	764	47.49	[19.5‒111.58]
More than 48 hours	71	29.30	[10.13‒152.51]

Moving forward, the ongoing evaluation will assess user satisfaction using the Net Promoter Score (NPS) scale and the impact of the new routine on the waiting time for the non-scheduled procedures. Continuous improvement initiatives will focus on reducing wait times, enhancing team collaboration, and refining decision-making processes to uphold the highest standards of patient care.

In conclusion, the adoption of a structured prioritization framework at HC-FMUSP represents a significant step towards mitigating resource constraints and enhancing patient care delivery. By fostering transparency, standardizing decision-making, and promoting interdisciplinary collaboration, the hospital has not only optimized surgical workflow but also improved patient outcomes. As healthcare evolves, integrating such innovative strategies will be crucial in meeting growing demands while maintaining quality and efficiency in surgical services.

By leveraging international best practices and tailoring them to local contexts, HC-FMUSP exemplifies proactive leadership in healthcare management, setting a benchmark for resource allocation strategies in complex hospital environments. This initiative emphasizes a systematic approach to prioritizing non-scheduled surgical procedures. It integrates theoretical frameworks, practical implementation steps, and preliminary outcomes, highlighting the transformative impact on hospital operations and patient care.

The successful implementation of this project sets a precedent for other institutions facing similar challenges, demonstrating the critical value of innovative and organized management strategies in healthcare.

## Declaration of competing interest

The authors declare no conflicts of interest.
